# Lightweight Quantum Authentication and Key Agreement Scheme in the Smart Grid Environment

**DOI:** 10.3390/e27090957

**Published:** 2025-09-14

**Authors:** Zehui Jiang, Run-Hua Shi

**Affiliations:** School of Control and Computer Engineering, North China Electric Power University, Beijing 102206, China; 120232227095@ncepu.edu.cn

**Keywords:** quantum communication, Bell state, smart grid, identity authentication, key agreement

## Abstract

Smart grids leverage smart terminal devices to collect information from the user side, achieving accurate load forecasting and optimized dispatching of power systems, effectively improving power supply efficiency and reliability while reducing energy consumption. However, the development of quantum technology poses severe challenges to the communication security of smart grids that rely on traditional cryptography. To address this security risk in the quantum era, this paper draws on the core idea of quantum private comparison and proposes a quantum-secure identity authentication and key agreement scheme suitable for smart grids. This scheme uses Bell states as quantum resources, combines hash functions and XOR operations, and can adapt to resource-constrained terminal devices. Through a security proof, it verifies the scheme’s ability to resist various attacks; the experimental results further show that the scheme still has good robustness in different noise environments, providing a feasible technical path for the secure communication of smart grids in the quantum environment and having clear practical engineering value.

## 1. Introduction

Since the early 20th century, as the theoretical framework of quantum physics has gradually been refined, unique properties such as quantum superposition and entanglement have continuously refreshed people’s understanding. The principle of quantum superposition allows quantum states to exist in superpositions of multiple states simultaneously, while quantum entanglement enables instantaneous correlations between two or more particles, even when they are separated by great distances, allowing them to influence each other. These revolutionary physical properties have not only propelled the development of fundamental science but have also brought about revolutionary changes in the field of information.

Quantum computing, leveraging the parallel computational power of quantum bits, can handle complex problems that are intractable for classical computers in a short amount of time. The Shor algorithm [1] can efficiently solve mathematical problems such as factorization and discrete logarithms, while the Grover algorithm [2] can double the speed of breaking symmetric encryption algorithms. Quantum computers can also utilize the Grover algorithm to attack hash functions, providing a square-root-level acceleration in finding collisions. Quantum communication, based on the principles of the no-cloning theorem and measurement collapse, can achieve theoretically unconditional secure information transmission. In 1984, Bennett and Brassard [3] proposed the first quantum key distribution (QKD) protocol, known as the BB84 protocol, which marked the beginning of a new era in the development of quantum communication. Since then, many new branches have emerged in the field of quantum communication, such as quantum secure direct communication [4,5], quantum secret sharing [6,7], and quantum private comparison [8,9].

In the classical domain, identity authentication is a crucial step in ensuring secure communication. Traditional identity authentication mechanisms based on cryptographic algorithms rely on computational complexity to ensure security, but they face the risk of being broken in the era of quantum computing. Therefore, researchers have begun to study identity authentication algorithms that can resist quantum attacks. This research is primarily divided into two directions: one direction involves the use of quantum-resistant encryption algorithms, such as lattice-based cryptography [10,11], and the other direction is to encrypt identity information using quantum states, which is known as quantum identity authentication schemes [12,13].

This paper focuses on the latter direction, which focuses on using quantum states to achieve identity authentication, particularly how to accomplish this goal with simple quantum operations and quantum states in resource-constrained scenarios. On one hand, simple quantum operations can significantly reduce the performance requirements of quantum hardware for the devices, thereby lowering hardware costs and size. On the other hand, by reducing the difficulty of preparing and measuring quantum states, the energy consumption can be substantially decreased, making the technology truly adaptable to practical environments in the future. The quantum resources used in this paper are Bell states, with quantum operations including Bell state measurements and Pauli operations. This article refers to the idea of quantum private comparison. Through the operation and measurement of quantum states, it compares whether the two communicating parties contain the same private information. We consider the possibility of applying this idea in the smart grid scenario and design a complete identity authentication and key agreement scheme for the communication network of the smart grid.

The smart grid is a novel power system based on information and communication technologies (ICT), designed to enhance the reliability, efficiency, and sustainability of the electricity supply [14]. Smart meters (SMs), as critical energy measurement devices within the smart grid, continuously monitor electricity usage, collect data, and transmit it to the smart grid management system. This facilitates precise energy metering, the implementation of dynamic pricing strategies, and the detection and localization of faults.

However, the data recorded by SMs is typically quite detailed, which includes information about specific devices and their usage times [15]. Eve can obtain users’ electricity consumption patterns and habits by eavesdropping on the data transmitted by SMs. This can lead to inference about the user’s lifestyle and routine activities, thereby infringing upon user privacy [16,17] and having adverse effects on the operation of the smart grid. Therefore, ensuring the legitimacy of identities between entities in smart grid communication and safeguarding user privacy from unauthorized access are of paramount importance [18,19]. The above classic schemes provide important paradigms for solving security authentication and communication issues in the smart grid environment. However, the development of quantum computing has had a huge impact on classical cryptography. Considering the context of the quantum era, the security of the above classic schemes needs to be re-evaluated.

To enable smart grids to meet the challenges of the quantum computing era, some researchers have considered introducing quantum communication technology into the smart grid environment. Their bold attempts have provided important ideas for subsequent researchers. Prateek et al. [20,21] have designed two quantum authentication and key management schemes for the smart grid, both based on QKD protocols. However, these schemes still face challenges such as resistance to entangle-measure attacks and high communication complexity. Parameswarath et al. [22] adopted a measurement-device-independent QKD (MDI-QKD) scheme. This is a QKD scheme that does not rely on any assumptions about measurement devices and has good scalability. However, this scheme still requires two untrusted entities to agree on a session key by sending quantum states to a trusted third party. Additionally, in deployment, quantum random number generators (QRNGs) are configured for each of the untrusted entities. Zhang et al. [23] chose a semi-QKD protocol, which realizes identity authentication through operations of selecting measurements or reflections based on a shared key. Nevertheless, it only considers the authentication between the control center (CC) and the gateway. Moreover, the secret information from the identity authentication phase is reused in the encryption process of message transmission. Therefore, in practical deployment, the protocol still needs to be extended. Considering the progressive nature of technology implementation, this paper attempts to combine quantum with classical approaches and abandons traditional symmetric encryption algorithms. In the processing of classical messages, the approach employs lightweight hash functions and XOR operations, which can theoretically enable quantum-secure authentication. Furthermore, the scheme proposed in this paper does not require SMs and gateways to have the capability to prepare quantum states, which is more in line with the issue of limited device resources in smart grid scenarios. The contributions of this paper are as follows:

(1) A secure authentication communication protocol suitable for the communication scenarios in the smart grid has been proposed. The protocol integrates hash functions with quantum communication, enabling identity authentication and the secure agreement of shared keys between the CC, gateways, and SMs.
(2) Although the key agreement between the two parties is assisted by a third party, the ultimately agreed-upon key is only secretly shared between the two parties, which greatly enhances the security of the scheme.
(3) We compared the anti-noise performance of the scheme in different noise environments on a simulation platform. Experiments have proved that our scheme has good performance under various types of noise, which provides a guarantee for the implementation of the scheme.

## 2. Preliminaries

### 2.1. Bell State

Bell states, also known as EPR pairs, are the simplest two-qubit entangled states and represent the maximum entanglement between two qubits. They form a complete orthogonal basis in the four-dimensional Hilbert space. There are four standard forms of Bell states, defined as follows [24]:

(1) $$\begin{array}{l} \left|\phi_{00}\rangle =\frac{1}{\sqrt{2}}(|00\rangle +|11\rangle ),|\phi_{01}\rangle =\frac{1}{\sqrt{2}}(|00\rangle -|11\rangle \right)\\ \left|\phi_{10}\rangle =\frac{1}{\sqrt{2}}(|01\rangle +|10\rangle ),|\phi_{11}\rangle =\frac{1}{\sqrt{2}}(|01\rangle -|10\rangle \right) \end{array}$$

The above four Bell states can all be rewritten into the following formula:

(2) $$\left|\phi_{ij}\rangle =\frac{1}{\sqrt{2}}(|0i\rangle +{\left(-1\right)}^{j}|1\bar{i}\rangle \right)$$

For the sake of convenience, we use the subscript “B=ij” or “M=ij” to refer to the specific Bell state during the calculation process.

### 2.2. Pauli Operation

Applying Pauli gates to qubits, which we are also accustomed to calling Pauli operations, is a common single-particle quantum gate operation. In the Pauli gates, X, Y, and Z are, respectively, called the X-gate, Y-gate, and Z-gate. In addition, we consider the identity gate operation I, which has no effect on the quantum state. These four Pauli operations can be represented in the form of the following quantum states [24].

(3) $$\begin{array}{l} U_{00}=I=|0\rangle \langle 0|+|1\rangle \langle 1|\\ U_{01}=\sigma_{z}=|0\rangle \langle 0|-|1\rangle \langle 1|\\ U_{10}=\sigma_{x}=|0\rangle \langle 1|+|1\rangle \langle 0|\\ U_{11}=i\sigma_{y}=|0\rangle \langle 1|-|1\rangle \langle 0| \end{array}$$

For the sake of convenience in representation, we use the subscript “ab” in $U_{ab}$ to refer to a specific Pauli operation during the calculation process.

### 2.3. Entanglement Swapping and Its Characteristics

**Figure 1:**
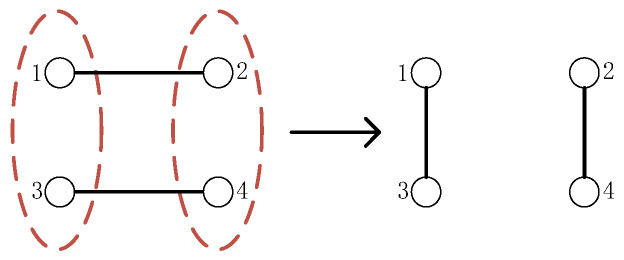
Entanglement swapping. The solid lines between the small balls represent the entanglement relationship between two particles, and the red dotted lines represent the Bell basis measurement.

Taking out some particles from each entangled state for quantum measurement will cause the measured particles and the unmeasured particles to collapse into two new entangled states. This is entanglement swapping. This paper only considers the entanglement swapping characteristics completed through Bell basis measurement between Bell states. The process is shown in Figure 1.

After the entanglement swapping of two Bell states through Bell basis measurement, two new Bell states will be generated. The state of the original Bell state and the state of the new Bell state satisfy the following equation relationship:

(4) $$M_{13}⊕M_{24}=B_{12}⊕B_{34}$$

We perform a Pauli operation on any one particle of a Bell state. The state of the newly generated Bell state satisfies the following equation:

(5) $${B_{12}}^{\prime }=B_{12}⊕a_{1}b_{1}$$

Therefore, when we first perform a Pauli operation on a Bell state and then perform entanglement swapping under the Bell basis, the state of the original Bell state and the state of the new Bell state satisfy the following equation:

(6) $$M_{13}⊕M_{24}=(B_{12}⊕B_{34})⊕(a_{1}b_{1}⊕a_{2}b_{2})$$

For the proof of the above equations, please refer to reference [25].

## 3. System Model and Security Model

In this section, we first introduce the system model in our solution, including the functions of various power grid entities and the simple communication process, as well as the basic assumptions in the model environment. Subsequently, we elaborate on the security model of this section from two perspectives: security objectives and threat model.

### 3.1. System Model

The smart grid model consists of SMs, gateways, and a CC. SMs are used to collect user side information and transmit power information to the CC through the aggregation function of gateways. The communication network of the smart grid has a hierarchical structure [26]. The user side area network is at the bottom layer and is composed of a variety of smart devices, and data is collected at the SMs. The data collected by SMs is aggregated at the gateways in the Neighborhood Area Network (NAN), and the aggregated data is submitted to the CC for data analysis. NAN gateways are intermediate nodes between SMs and the CC and also act as forwarding relays. Any message transmission between SMs and the CC, including classical messages and quantum messages, needs to pass through the transfer of NAN gateways. Therefore, it is necessary for the CC to conduct legitimacy inspection on their identities. Meanwhile, as an aggregation node of SMs, the NAN gateway needs to communicate securely with SMs. Thus, it is necessary to agree on a security key for an SM and a NAN gateway. In addition, in this paper, the CC is secure and trustworthy. It not only needs to be responsible for the identity authentication processing of SMs and NAN gateways but also needs to handle their registration requests. Meanwhile, the CC has complete quantum capabilities. Later, in the key agreement process between SMs and NAN gateways, it acts as an auxiliary third party to distribute necessary quantum states for both parties.

In addition, we require that clock synchronization be maintained among various devices in the smart grid environment. Finally, some classical information needs to be announced during the execution of the scheme. We assume that there is a broadcast channel. In reality, this can be a bulletin board or a blockchain.

**Figure 2:**
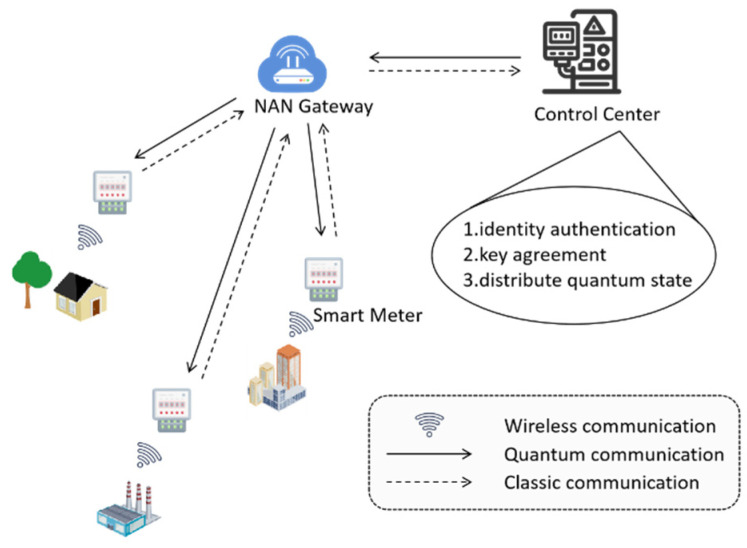
System model.

The specific model structure is shown as follows in Figure 2.

### 3.2. Security Model

Security Objectives: The CC needs to complete the identity authentication of NAN gateways and SMs and needs to assist SMs and NAN gateways with legitimate identities in completing the agreement of session keys.

Threat Model: (1) The data transmitted by SMs contains a large amount of privacy-related information. Therefore, it is very likely to be attacked by malicious actors during network communication. Common attacks include eavesdropping attacks, man-in-the-middle attacks, replay attacks, etc. In addition, for the quantum communication process, we need to assume that the attacker has complete quantum capabilities and can carry out attack methods such as entangle-measure attacks. (2) Finally, the scheme not only needs to be able to prevent attacks from external attackers but also needs to ensure that even participants can only obtain necessary information. For example, in the key agreement process, although the CC, as an auxiliary third party, participates in this process, it still needs to ensure that the key is only shared between the SM and the NAN gateway.

## 4. Scheme

The entire scheme is divided into three phases: (1) Registration Phase; (2) Identity Authentication Phase; (3) Key Agreement Phase. Initially, the CC, NAN gateways, and SMs complete the initial deployment. The CC, as a secure and trusted institution, receives the registration requests from NAN gateways and SMs. Before the official communication, NAN gateways and SMs need to authenticate their legitimate identities to the CC. After the authentication is completed, an SM can, with the assistance of the CC, agree on a session key with a NAN gateway. The symbols used in the protocol and their explanations are shown in Table 1.

### 4.1. Registration Phase

In each stage of communication, various security risks may exist. However, in the registration phase, we assume that it is carried out under absolutely secure conditions. This stage mainly completes the initialization of the system, including the announcement of public parameters and the distribution of private information. The CC publishes a hash function family H. The specific hash function in this hash function family [27] is selected through a key. That is to say, different keys can locate different hash functions, and without obtaining the key, one cannot obtain the hash function of others. There is a secure master key $K_{CC}$ in the CC, and the CC can locate the hash function $h_{K\sim cc}$ from H.

In addition, in this phase, the NAN gateway uses its own identity information ${ID}_{NAN}$ and location information to register with the CC. The CC will check the uniqueness of this ID. If the ID has been registered, the request will be rejected. Otherwise, the CC will issue a master key $K_{NAN}$, a key stream function $F_{NAN}(\cdot )$, and a random seed $R_{NAN}$ to the NAN gateway. Different NAN gateways correspond to different key stream functions, and this function can generate different key streams with different seeds as parameters. Finally, the CC announces the existence of the NAN gateway and publishes its location on the bulletin board or in the blockchain.

The SM selects an appropriate NAN gateway according to its access location and carries the identity information ${ID}_{SM}$ to register with the CC. The CC also checks the uniqueness of this ID. If the ID has been registered, the request will be rejected. Otherwise, the CC generates a random number ${rand}_{SM}$ for it and generates a pseudonym ${PID}_{SM}=h_{K\sim cc}({ID}_{SM}||{rand}_{SM})$. According to the NAN gateway information reported by the SM, the CC finds the corresponding key stream function $F_{NAN}(\cdot )$, and issues this key stream function to the SM, including a master key $K_{SM}$ and a random seed $R_{SM}$.

After receiving the information issued by the CC, the NAN gateway and the SM each store it in their local databases.

### 4.2. Identity Authentication Phase

Each NAN gateway is equivalent to a supervisor, managing all SMs in a certain area and completing the forwarding of SM information. Therefore, before the CC verifies the legitimacy of the identity of this SM, it needs to first verify the identity of the NAN gateway in this area.

**Step 1-1:** The SM sends an identity verification message ${Req}_{SM}={PID}_{SM}||{TS}_{1}$ to the NAN gateway in its area. After receiving the message, the NAN gateway forwards the message to the CC and initiates another identity verification request ${Req}_{NAN}={ID}_{NAN}||{TS}_{2}$ to the CC.
**Step 1-2:** The CC and the NAN gateway use the master key $K_{NAN}$ to select a hash function $h_{K\sim NAN}$ from H. Use this hash function to calculate $h_{K\sim NAN}({ID}_{NAN}||{TS}_{2})$. In order to further reduce the likelihood of a collision occurring, the CC and the NAN gateway use $K_{NAN}$ as the seed of the key stream function to, respectively, generate an n-bit key $K_{OTP\sim NAN}$. The CC and the NAN gateway calculate $h_{K\sim NAN}({ID}_{NAN}|\left|{TS}_{2}\right)⨁K_{OTP\sim NAN}$. Suppose the XOR values obtained by the CC and the NAN gateway are ${Privacy}_{0}$ and ${Privacy}_{1},$ respectively.
**Step 1-3:** The CC randomly generates n Bell states and pairs the n Bell states two by two. Suppose the two Bell states are ${|\left.\phi \right\rangle }_{12}$ and ${|\left.\phi \right\rangle }_{34}$. The CC splits the Bell states into 13 and 24 sequences, retains the 13 sequence by itself, and sends the 24 sequence to the NAN gateway through the quantum channel. Assume the sent quantum sequence is Q. To check whether there is an eavesdropper during the transmission process, the CC randomly generates n/2 decoy photons and inserts these qubits into the quantum sequence transmitted to the NAN gateway. The generation rules of the decoy photons are as follows.

The CC and the NAN gateway use the master key $K_{NAN}$ to generate n integers through a cyclic left shift. The generation rule is shown in Table 2 (taking n = 6 and $K_{NAN}=\;011010$ as an example). $K_{NAN}$ can be expressed as $K_{NAN}=\left\{k_{N1},k_{N2},k_{N3},k_{N4},k_{N5},k_{N6}\right\}=\{26,52,41,19,38,13\}\;mod(n\;+\;1)=\{5,3,6,5,3,6\}$. The NAN gateway announces a random permutation function π(·) [28] using its ID, and the CC obtains $\pi \left(K_{NAN}\right)=K_{NAN}^{\prime }=\left\{k_{\pi (N1)},k_{\pi (N2)},k_{\pi (N3)},k_{\pi (N4)},k_{\pi (N5)},k_{\pi (N6)}\right\}$ by executing the permutation function $\pi \left(K_{NAN}\right)$.

**Figure 3:**
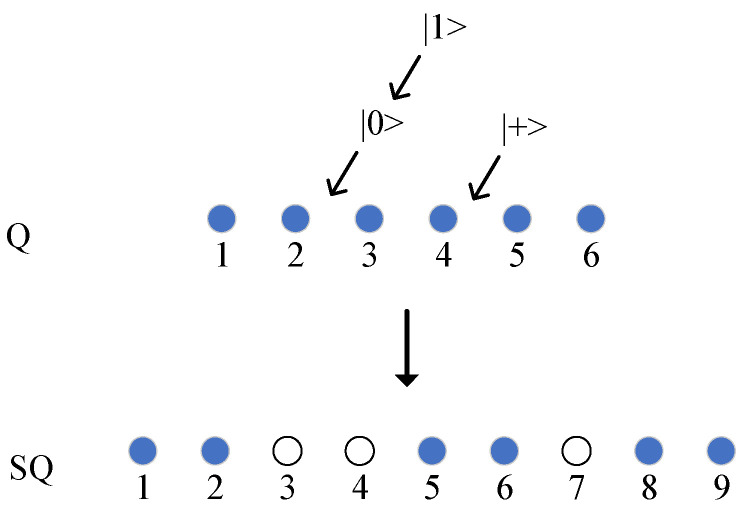
Decoy photon insertion, with hollow small balls serving as the inserted decoy photons.

The CC generates n/2 decoy photons, and each quantum state is randomly selected from $\{|\left.0\right\rangle ,|\left.1\right\rangle ,|\left.+\right\rangle ,|\left.-\right\rangle$; for example, $\left\{|\left.0\right\rangle ,|\left.+\right\rangle ,|\left.1\right\rangle \right\}$ is selected. The CC inserts the decoy photons into Q according to the first n/2 bits of $K_{NAN}^{\prime }$ to obtain the sequence SQ. Assuming $K_{NAN}^{\prime }=\{3,5,3,6,6,5\}$, the insertion result is shown in Figure 3. The CC records the decoy photons of each insertion position and sends the sequence SQ to the NAN gateway.

**Steps 1-4:** When the NAN gateway receives the particles at the position corresponding to the decoy photons, it randomly selects the Z-basis $\left\{|\left.0\right\rangle ,|\left.1\right\rangle \right\}$ or the X-basis $\left\{|\left.+\right\rangle ,|\left.-\right\rangle \right\}$ to directly measure the particles. After the NAN gateway receives all particles, it requires the CC to announce the states of the decoy photons at the corresponding position. The NAN gateway compares its measurement result under the correct basis with the result announced by the CC. If the error rate is lower than a certain threshold, the NAN gateway successfully authenticates the identity of the CC and proceeds to the next step. Otherwise, it is considered that there is eavesdropping, and the protocol is terminated. It should be noted that to prevent an attacker from impersonating the CC, if all measurement results of the NAN gateway under the correct basis are the same, the authentication process is invalid, and it is considered that there is an eavesdropper. If it is considered that there is an eavesdropper, the detecting party needs to broadcast a reminder message to tell the other party that there may be an eavesdropper. At this time, the CC and the NAN gateway need to use the same method to synchronously update the master key $K_{NAN}$ and announce a new permutation function. Subsequently, the operation of inserting decoy states for eavesdropping detection is referred to as “inserting decoy photons and performing eavesdropping detection”.
**Steps 1-5:** This step is carried out synchronously with Steps 1-4. The CC and the NAN gateway sequentially select two bits from ${Privacy}_{0}$ and ${Privacy}_{1}$ in order. The CC can select a Pauli operation based on this and apply it to any one of the two particles in the corresponding order. However, to improve the protocol execution speed, the NAN gateway will apply this Pauli operation to the first particle of the paired two particles and directly perform Bell basis measurement after the second particle arrives. For example, if the two bits selected by the NAN gateway at this time are 01, it is equivalent to the NAN gateway performing the operation $\left(U_{01}⨂I\right){|\left.\phi \right\rangle }_{24}$ on the two particles in its hand.
**Steps 1-6:** After the CC and the NAN gateway complete the Bell basis measurement of the particles in their hands, they obtain measurement results $\left\{M_{13},M_{57},\ldots \ldots ,M_{2n-3,2n-1}\right\}$ and $\left\{M_{24},M_{68},\ldots \ldots ,M_{2n-2,2n}\right\},$ respectively. The NAN gateway announces its own measurement results.
**Steps 1-7:** The CC calculates $M_{13}⨁M_{24}⨁B_{12}⨁B_{34}$ and calculates subsequent results in sequence. If the final result is all 0s, the CC indicates that the identity authentication of the NAN gateway is successful; otherwise, the CC discards the identity authentication request for the NAN gateway and terminates the protocol. After verifying the legitimacy of the identity of the NAN gateway, the CC starts to verify the identity of the SM through the forwarding of the NAN gateway. This process is similar to the above-mentioned process, and the specific process is as follows.
**Steps 1-8:** The CC and the SM use the master key $K_{SM}$ to select a hash function $h_{K\sim SM}$ from H. Use the hash function to calculate $h_{K\sim SM}({PID}_{SM}||{TS}_{1})$. Meanwhile, the CC and the SM use $K_{SM}$ as the seed of the key stream function to, respectively, generate an n-bit key $K_{OTP\sim SM}$. The CC and the SM calculate $h_{K\sim SM}(P{ID}_{SM}|\left|{TS}_{1}\right)⨁K_{OTP\sim SM}$. Suppose the XOR values obtained by the CC and the SM are ${Privacy}_{2}$ and ${Privacy}_{3},$ respectively.
**Steps 1-9:** The CC randomly generates n Bell states and pairs the n Bell states two by two. Split them into two sequences according to the splitting method in Steps 1-3. The CC retains one sequence by itself and sends the other sequence to the SM and acts “inserting decoy photons and performing eavesdropping detection”.
**Steps 1-10:** According to the rule in Steps 1-5, the CC and the SM complete the Pauli operation and Bell basis measurement on the particles each holds, and the SM announces its measurement result.
**Step 1-11:** The CC calculates the legitimacy of the identity of the SM based on the measurement results of both parties and the initial state of the distributed Bell states. If it is not legal, the CC will reject the request of the SM and discard the identity verification message.

### 4.3. Key Agreement Phase

**Step 2-1:** After the identity of the SM is verified, the CC generates a random seed $R_{session}$ for the NAN gateway and the SM. The CC uses $R_{SM}$ to generate an l-bit $K_{OTP\sim SM}^{\prime }$, calculates ${ACKReq}_{SM}=K_{OTP\sim SM}^{\prime }⨁{ACK}_{SM}$, and sends it to the SM, where ${ACK}_{SM}={PID}_{SM}||R_{session}||{TS}_{3}$. The SM decrypts to obtain ${ACK}_{SM}$, verifies that the message indeed comes from the CC by checking ${PID}_{SM}$, and accepts the random seed $R_{session}$. Let the total length of ${PID}_{SM}||R_{session}||{TS}_{3}\;$ be l.
**Step 2-2:** The CC uses the random seed $R_{NAN}$ to generate an l-bit $K_{OTP\sim NAN}^{\prime }$ for encryption, obtains ${ACKReq}_{NAN}=K_{OTP\sim NAN}^{\prime }⨁{ACK}_{NAN}$, and sends it to the NAN gateway, where ${ACK}_{NAN}={ID}_{NAN}||R_{session}||{TS}_{4}$. After decryption, the NAN gateway obtains $R_{session}$. Let the total length of $R_{session}||{TS}_{4}$ be l.
**Step 2-3:** The CC randomly generates k Bell states $\left\{{|\left.\phi \right\rangle }_{12},{|\left.\phi \right\rangle }_{34},\ldots \ldots {,|\left.\phi \right\rangle }_{2k-1,2k}\right\}$, k is even, splits them into two sequences according to the splitting method in Step 1-3, sends them to the NAN gateway and the SM, respectively, and acts “inserting decoy photons and performing eavesdropping detection”.
**Step 2-4:** The CC announces the original states of k Bell states. Meanwhile, the NAN gateway and the SM generate a k-bit string sequence $\left\{r_{1},r_{2},\ldots \ldots {,r}_{k}\right\}$ with the help of $R_{session}$. The NAN gateway calculates $k_{session}=M_{13}^{\prime }⨁B_{12}^{\prime }⨁r_{1}r_{2}$, and the SM calculates $k_{session}=M_{24}^{\prime }⨁B_{34}^{\prime }⨁r_{1}r_{2}$, which are used as the session keys for the NAN gateway and the SM. By analogy, the NAN gateway and the SM calculate all the XOR values. Finally, the NAN gateway and the SM obtain the session key $K_{session}$ with each other.

## 5. Analysis

In this section, we analyze the security of the proposed scheme in this paper, mainly including the scheme’s resistance to external and internal attacks. Finally, we compare the scheme in this paper with other schemes.

### 5.1. Quantum Security of the Quantum Communication Process

In this subsection, we analyze the quantum security in the process of quantum communication. The scheme executes the protocol by distributing Bell states in the identity authentication phase and the key agreement phase, respectively. We assume that the input state of the quantum system in this process is $\rho_{in}$ and the output state is $\rho_{out}$. If for each input state, the output state is a completely mixed state, then we consider this process to be quantum-secure [29]. Among them, the input state and the output state satisfy the following equation:

(7) ρout=∑ipiUiρinUi=†12nI

Here, $\rho_{in}$ is the density matrix of n-bit all possible input states, and $U_{i}$ is the corresponding unitary operation performing on the input state.

During the execution of the protocol, assume that the two particles of the entangled Bell state distributed by the CC in a certain time are (t,h). For the sake of clear expression, we only analyze the distributed particle (t). Correspondingly, we can obtain

(8) $$\begin{array}{l} \rho_{in}(t)=Tr_{h}(\rho_{th})\\ =Tr_{h}(\frac{1}{4}{|\phi^{+}\rangle }_{th}\langle \phi^{+}|+\frac{1}{4}{|\phi^{-}\rangle }_{th}\langle \phi^{-}|+\frac{1}{4}{|\psi^{+}\rangle }_{th}\langle \psi^{+}|+\frac{1}{4}{|\psi^{-}\rangle }_{th}\langle \psi^{-}|)\\ =(\frac{1}{4}{|0\rangle }_{t}\langle 0|+\frac{1}{4}{|1\rangle }_{t}\langle 1|+\frac{1}{4}{|+\rangle }_{t}\langle +|+\frac{1}{4}{|-\rangle }_{t}\langle -|)\\ =\frac{1}{4}\left(\left(\begin{array}{cc} 1 & 0\\ 0 & 0 \end{array}\right)+\left(\begin{array}{cc} 0 & 0\\ 0 & 1 \end{array}\right)+\frac{1}{2}\left(\begin{array}{cc} 1 & 1\\ 1 & 1 \end{array}\right)+\frac{1}{2}\left(\begin{array}{cc} 1 & -1\\ -1 & 1 \end{array}\right)\right)=\frac{1}{2}\left(\begin{array}{cc} 1 & 0\\ 0 & 1 \end{array}\right)=\frac{I}{2} \end{array}$$

When the party receiving the quantum performs a Pauli operation according to its own private information, the output state of particle t is

(9) ρout(t)=Trh[14(I(14|ϕ00〉th〈ϕ00|+14|ϕ01〉th〈ϕ01|+14|ϕ10〉th〈ϕ10|+14|ϕ11〉th〈ϕ11|)I†)+14(σz(14|ϕ00〉th〈ϕ00|+14|ϕ01〉th〈ϕ01|+14|ϕ10〉th〈ϕ10|+14|ϕ11〉th〈ϕ11|)σz)†+14(σx(14|ϕ00〉th〈ϕ00|+14|ϕ01〉th〈ϕ01|+14|ϕ10〉th〈ϕ10|+14|ϕ11〉th〈ϕ11|)σx)†+14(iσy(14|ϕ00〉th〈ϕ00|+14|ϕ01〉th〈ϕ01|+14|ϕ10〉th〈ϕ10|+14|ϕ11〉th〈ϕ11|)iσy)†]=Trh(14|ϕ00〉th〈ϕ00|+14|ϕ01〉th〈ϕ01|+14|ϕ10〉th〈ϕ10|+14|ϕ11〉th〈ϕ11|)=14[|0〉t〈0|+|1〉t〈1|+|+〉t〈+|+|−〉t〈−|]=141000+0001+121111+121−1−11=121001=I2

We know from Equation (9) that the output state of the particle is a completely mixed state. Therefore, this process is quantum-secure.

### 5.2. External Security

#### 5.2.1. Entangle-Measure Attack

Our scheme can effectively resist entangle-measure attacks. We assume that whether the CC distributes part of the Bell states during the identity authentication process or distributes all of them during the key agreement process, Eve can intercept all the particles. The unitary operation operator implemented by Eve is U, and $|\left.au\right\rangle$ is Eve’s auxiliary particle. When Eve’s unitary operation acts on the decoy photons $|\left.0\right\rangle$ and $|\left.1\right\rangle$, it can be expressed as

(10) $$\begin{array}{l} U|0\rangle |au\rangle =\lambda_{0}|0\rangle |e_{00}\rangle +\lambda_{1}|1\rangle |e_{01}\rangle \\ U|1\rangle |au\rangle =\lambda_{2}|0\rangle |e_{10}\rangle +\lambda_{3}|1\rangle |e_{11}\rangle \end{array}$$

Among them, $|\left.e_{00}\right\rangle ,|\left.e_{01}\right\rangle ,|\left.e_{10}\right\rangle ,|\left.e_{11}\right\rangle \;$ are the pure states generated after the action of U. The coefficients of the above equations satisfy

(11) $${\left|\lambda_{0}\right|}^{2}+{\left|\lambda_{1}\right|}^{2}=1,{\left|\lambda_{2}\right|}^{2}+{\left|\lambda_{3}\right|}^{2}=1$$

Therefore, it can be obtained that when the decoy photons are $\left|\left.+\right\rangle =\frac{1}{\sqrt{2}}(|\left.0\right\rangle +|\left.1\right\rangle \right)$ and $\left|\left.-\right\rangle =\frac{1}{\sqrt{2}}(|\left.0\right\rangle -|\left.1\right\rangle \right)$, they can be expressed as

(12) $$\begin{array}{l} U|+\rangle |au\rangle =\frac{1}{2}|+\rangle (\lambda_{0}|e_{00}\rangle +\lambda_{1}|e_{01}\rangle +\lambda_{2}|e_{10}\rangle +\lambda_{3}|e_{11}\rangle )\\ +\frac{1}{2}|-\rangle (\lambda_{0}|e_{00}\rangle -\lambda_{1}|e_{01}\rangle +\lambda_{2}|e_{10}\rangle -\lambda_{3}|e_{11}\rangle )\\ U|-\rangle |au\rangle =\frac{1}{2}|+\rangle (\lambda_{0}|e_{00}\rangle +\lambda_{1}|e_{01}\rangle -\lambda_{2}|e_{10}\rangle -\lambda_{3}|e_{11}\rangle )\\ +\frac{1}{2}|-\rangle (\lambda_{0}|e_{00}\rangle -\lambda_{1}|e_{01}\rangle -\lambda_{2}|e_{10}\rangle +\lambda_{3}|e_{11}\rangle ) \end{array}$$

We see from Equations (10) and (12) that when the following conditions are met,

(13) $$\lambda_{1}=\lambda_{2}=0,\lambda_{0}=\lambda_{3}=1,|e_{00}\rangle =|e_{11}\rangle$$

Eve can avoid being detected in the eavesdropping detection. Substituting (17) into (14) and (16) shows that

(14) $$\begin{array}{l} U|0\rangle |au\rangle =|0\rangle |e_{00}\rangle \\ U|1\rangle |au\rangle =|1\rangle |e_{11}\rangle \end{array}$$

(15) $$\begin{array}{l} U|+\rangle |au\rangle =\frac{1}{2}|+\rangle (|e_{00}\rangle +|e_{11}\rangle )\\ U|-\rangle |au\rangle =\frac{1}{2}|-\rangle (|e_{00}\rangle +|e_{11}\rangle ) \end{array}$$

Thus, when acting on the Bell state, we have

(16) $$\begin{array}{l} U_{1}⊗U_{2}|\phi_{ij}\rangle |au\rangle_{1}|au\rangle_{2}\\ =U_{1}⊗U_{2}\frac{1}{\sqrt{2}}(|0i\rangle +{\left(-1\right)}^{j}|1\bar{i}\rangle )|au\rangle_{1}|au\rangle_{2}\\ =\frac{1}{\sqrt{2}}(U_{1}⊗U_{2}|0i\rangle |au\rangle_{1}|au\rangle_{2}+U_{1}⊗U_{2}{\left(-1\right)}^{j}|1\bar{i}\rangle |au\rangle_{1}|au\rangle_{2})\\ =\frac{1}{\sqrt{2}}(U_{1}|0\rangle |au\rangle_{1}⊗U_{2}|i\rangle |au\rangle_{2}+U_{1}|1\rangle |au\rangle_{1}⊗U_{2}{\left(-1\right)}^{j}|\bar{i}\rangle |au\rangle_{2})\\ =\frac{1}{\sqrt{2}}(|0i\rangle |e_{00}\rangle |e_{ii}\rangle +{\left(-1\right)}^{j}|1\bar{i}\rangle |e_{11}\rangle |e_{\bar{i}\bar{i}}\rangle )=|\phi_{ij}\rangle |e_{00}\rangle |e_{ii}\rangle \end{array}$$

From Equation (16), when the auxiliary particle and the intercepted particle are in a direct product state, Eve’s entangle-measure attack will not be detected. However, due to the insertion of decoy photons, Eve cannot know exactly which position the two entangled particles are in, so Eve cannot effectively carry out the entangle-measure attack.

#### 5.2.2. Replay Attack

A replay attack refers to an attacker intercepting information of legitimate users and replaying the intercepted messages within subsequent time to deceive the system into conducting legitimate activities including identity authentication. However, the scheme in this paper can effectively resist replay attacks.

First, in the identity authentication phase, an attacker may attempt to replay the messages of SMs and NAN gateways to deceive the CC, and the messages that the attacker can use for replay include identity authentication request messages and messages of measurement results of Bell states under the Bell basis. It should be noted that since the authentication request contains a timestamp, the CC will discard expired requests. Even if the attacker modifies the timestamp, the following problems will occur:

(1) The privacy-related information generated by the CC for comparison depends on the master key $K_{X}$ generated each time. Attackers cannot generate privacy information identical to that of the CC without obtaining the master key.
(2) The quantum states of the Bell states generated by the CC each time are random. Therefore, the attacker cannot deceive the CC by replaying the measurement results of SMs and NAN gateways. Otherwise, when the CC conducts privacy comparison, the calculation result can only be all 0s with a probability close to 0, which is equivalent to the attacker randomly announcing the measurement results.

Even in the key agreement phase, it is impossible for Eve to deceive SMs or NAN gateways by replaying the messages of the CC, because SMs and NAN gateways will not continuously use the same key stream for decryption.

#### 5.2.3. Eavesdropping Attack

From the above analysis, we can see that it is meaningless for an attacker to eavesdrop on the classical messages transmitted between entities in the power grid, and no information can be obtained. Therefore, an attacker may eavesdrop on quantum messages to steal secrets. However, the scheme in this paper can effectively resist eavesdropping attacks.

When distributing quantum sequences, the CC will insert a certain number of decoy photons. Because of the existence of decoy photons, the CC will conduct eavesdropping detection with NAN gateways or SMs. When an eavesdropper eavesdrops on quantum information, he will perform measurements first. Since the eavesdropper randomly selects the measurement basis, for each quantum state, the eavesdropper only has a 1/2 probability of selecting the correct measurement basis. For the case of wrong selection, when NAN gateways or SMs perform measurements according to the positions and states announced by the CC, the wrong quantum state will have a 1/2 probability of collapsing to the originally correct quantum state. Therefore, the probability that the eavesdropper escapes being detected is 1/2+1/2×1/2=3/4, and the probability of being detected is 1−3/4=1/4. When the number of added decoy photons is p, the probability that the eavesdropper escapes being detected is ${\left(3/4\right)}^{p}$. When p=15, the probability that the eavesdropper escapes being detected is only 1.34%, and in actual transmission, the value of p is much larger than 15.

#### 5.2.4. Man-in-the-Middle Attack (MITM)

Due to the fact that in the communication model of this paper, NAN gateways, as intermediate nodes, are responsible for forwarding messages between SMs and the CC, the positions where an attacker can carry out MITMs can only be between SMs and NAN gateways, and between NAN gateways and the CC. However, the scheme of this paper can effectively resist MITMs.

Consider an attacker carrying out a MITM between a SM and a NAN gateway. First, in the identity authentication phase, the communication between the SM and the NAN gateway includes the SM sending an authentication request and the NAN gateway forwarding the quantum states sent by the CC. From the above analysis, even if the attacker uses attack means such as forging or tampering with the authentication request, as well as entanglement measurement and measurement eavesdropping, they cannot obtain valid information or deceive the system. Secondly, in the key agreement phase, the communication between the SM and the NAN gateway includes the response messages and quantum states forwarded by the NAN gateway from the CC. However, the prerequisite for an attacker to effectively forge or tamper with response messages at this stage is that the attacker can obtain the random seed $R_{SM}$, which is generated during the registration phase. The attacker cannot obtain it. Similarly, the attacker cannot attack the quantum information.

Consider an attacker carrying out a MITM between a NAN gateway and the CC. The means for the attacker to carry out the MITM here are similar to those above, and the messages exchanged between the NAN gateway and the CC are similar to those exchanged between the SM and the NAN gateway. The analysis process refers to the above analysis.

### 5.3. Internal Security

In the identity authentication phase, during the process of the SM authenticating its identity to the CC, except for the need for the NAN gateway to forward information and quantum states, encryption operations and measurement operations by the NAN gateway are not required. Therefore, the NAN gateway cannot obtain the privacy-related information of the SM. Similarly, the SM cannot obtain the privacy-related information of the NAN gateway. In the key agreement phase, the NAN gateway cannot decrypt the information that needs to be forwarded by it under the premise that it only has its own privacy-related information. Therefore, the security analysis of internal attacks carried out by SMs and NAN gateways is equivalent to the security analysis of external attacks. So, in this subsection, we only discuss the ability of the CC to obtain the session keys of SMs and NAN gateways in the key agreement phase.

In the key agreement phase, the NAN gateway and the SM obtain the key by calculating $M_{13}^{\prime }⨁B_{12}^{\prime }⨁r_{1}r_{2}$ and $M_{24}^{\prime }⨁B_{34}^{\prime }⨁r_{1}r_{2}$ respectively. Because the CC is responsible for distributing Bell states and generating the random seed $R_{session}$. Therefore, the CC is capable of knowing $B_{12}^{\prime }$, $B_{34}^{\prime }$ and $r_{1}r_{2}$. According to the relationship in Equation (4), it can be obtained that

(17) $$M_{13}^{\prime }⊕B_{12}^{\prime }=M_{24}^{\prime }⊕B_{34}^{\prime }$$

Therefore, NAN gateways and SMs can obtain the same key. Assume that at a certain moment, the two Bell states generated by the CC are both $|\left.\phi_{00}\right\rangle$. For the convenience of representation, we use ${|\left.\phi^{+}\right\rangle }_{12}$ and ${|\left.\phi^{+}\right\rangle }_{34}\;$ to represent them. $|\left.\phi_{00}\right\rangle$ and $|\left.\phi^{+}\right\rangle$ represent the same Bell state, and the subscripts 1, 2, 3, and 4 represent different particles in the two Bell states. Then, the entanglement swapping process between them can be represented as

(18) $$\begin{array}{l} |\phi^{+}\rangle_{12}⊗|\phi^{+}\rangle_{34}=\frac{1}{2}(|00\rangle +|11\rangle {)}_{12}⊗(|00\rangle +|11\rangle {)}_{34}\\ \to \frac{1}{2}{(|00\rangle }_{13}|00\rangle_{24}+|01\rangle_{13}|01\rangle_{24}+|10\rangle_{13}|10\rangle_{24}+|11\rangle_{13}|11\rangle_{24})\\ =\frac{1}{2}{(|\phi^{+}\rangle }_{13}|\phi^{+}\rangle_{24}+|\phi^{-}\rangle_{13}|\phi^{-}\rangle_{24}+|\varphi^{+}\rangle_{13}|\varphi^{+}\rangle_{24}+|\varphi^{-}\rangle_{13}|\varphi^{-}\rangle_{24}) \end{array}$$

From Equation (18), even if the CC backs up the Bell states and performs entanglement swapping measurement in the Bell basis before distributing the Bell state sequences, the measurement result will also be a random one among the four Bell states. That is to say, this key agreement process ensures that NAN gateways and SMs can obtain the same result when calculating the shared key, but which specific result is random and unknown. Therefore, even the CC cannot obtain any information about the shared key.

### 5.4. Comparison

In this subsection, we will analyze the scheme from aspects such as quantum efficiency, quantum operations, classical operations, and the quantum capabilities of participants, and compare it with other existing schemes, as shown in Table 3. Quantum efficiency is an important indicator for measuring quantum communication protocols and can be defined [8] as $e=\frac{c}{t}$. Here, c represents the number of classical bits to be authenticated, and t represents the total number of quantum bits to be consumed. When we count t, decoy states are not considered at all. We regard the use of decoy states for eavesdropping detection as a public step. In this paper, each entanglement swapping involves 2 Bell states, that is, 4 quantum bits, and can compare or agree on 2 classical bits of information. Therefore, the quantum efficiency of the scheme in this paper is 50%.

In comparison with other schemes, Ref. [21] requires NAN gateways and SMs to continuously generate quantum resources, which is undoubtedly a huge challenge for grid devices with limited resources. Ref. [20] adopted a semi-quantum protocol to improve it. However, in the key agreement phase, it generates quantum bits nearly 9 times the number of classical bits. Moreover, this scheme attempts to combine Bell states and single photons, which actually brings great difficulties to the manufacturing of internal quantum devices of the equipment. Ref. [22] realizes authentication and key update through QKD + QRNG. In the stage of selecting the measurement basis in QKD, 50% of the quantum states are discarded, so the overall quantum efficiency is approximately equal to 50%. Ref. [30] improved the quantum efficiency of identity authentication to 100%. However, the article uses GHZ states to achieve batch authentication. To authenticate n devices, it is necessary to prepare GHZ states with n+1 particle entanglements, which brings an exponential-level difficulty to the preparation of quantum states. We believe that it is almost impossible to implement in an actual environment.

## 6. Experiment

Under the existing quantum technology, noise is one of the main challenges faced by quantum systems. It will significantly affect the coherence of quantum bits, the fidelity of gate operations, and the scalability of the overall system. Therefore, adding the simulation of noise to quantum circuits can help us find quantum algorithms that are robust to noise or design quantum schemes that reduce the impact of noise. The mathematical model of noise on the quantum system can be defined as:

(19) $$\varepsilon (\rho )=\sum_{k=0}^{m-1}E_{k}\rho E_{k}^{†}$$

where $E_{k}$ is the Kraus operator, ρ is the density matrix of the quantum state, and ε(ρ) is the evolved quantum state. For common types of noise, such as amplitude damping noise, phase damping noise, bit-flip noise, phase-flip noise, and depolarizing noise, their Kraus operators are summarized in Table 4.

Designing a reasonable quantum scheme can, to a certain extent, alleviate the overall impact of quantum noise on the system. For example, when comparing with single-quantum protocols, the same intensity of bit-flip noise may bring about different degrees of impact. The role of bit-flip noise is to flip the state of a quantum bit. It can turn $\left.|0\right\rangle$ into $\left.|1\right\rangle$ and $\left.|1\right\rangle$ into $\left.|0\right\rangle$. In a single-quantum protocol, the flip of each quantum bit means an error in the measurement of that quantum bit. However, in the scheme of this paper, assume that the two Bell states for an entanglement swapping are ${|\left.\phi \right\rangle }_{12}=\frac{1}{\sqrt{2}}(|\left.00\right\rangle +|\left.11\right\rangle )$ and ${|\left.\phi \right\rangle }_{34}=\frac{1}{\sqrt{2}}(|\left.01\right\rangle +|\left.10\right\rangle )$ respectively. The bit-flip noise just affects both particles 1 and 3. The two Bell states after the noise impact do not have any impact on the system results when performing Bell basis measurement. This is also an important reason why the scheme of this paper can operate normally in a certain noise environment. We compared the performance of the scheme of this paper with that of single-quantum bits in the same noise environment. We set the value range of the experimental noise probability p as [0, 0.3] with a step size of 0.02. In the scheme, we perform Bell basis measurement on every two quantum bits as a group. For the single-quantum scheme, the probability that both qubits can ensure correctness is:

(20) $$p_{true}=1-2p+p^{2}$$

**Figure 4:**
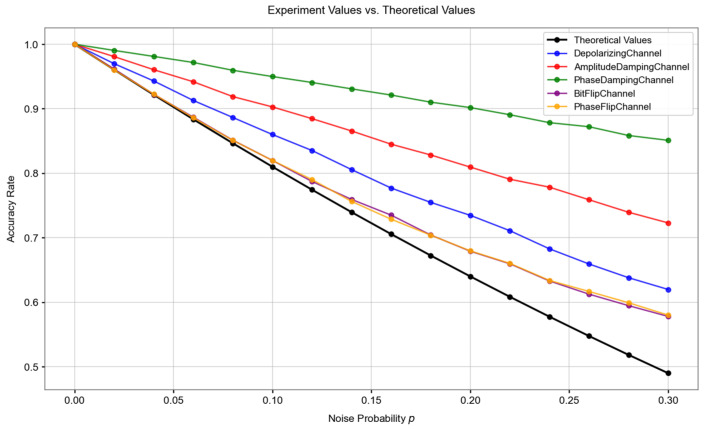
Comparison of the experimental correct probability and the theoretical correct probability results under different noise environments.

We take this probability as the theoretical value of the probability that the scheme can still ensure correctness in a noisy environment. The results of the experimental correct probability and the theoretical correct probability under different noise environments are shown in the following Figure 4.

The experimental results shown in the above figure indicate that the scheme can exhibit a certain degree of robustness under different quantum noise environments. Assuming that we take a 10% correct rate as the threshold, the single-quantum scheme faces the risk of failure when the noise probability is approximately 0.05. However, the scheme in this paper can maintain a correct rate higher than the threshold under a wider range of noise probabilities. Even if the noise intensity gradually increases, its performance degradation is more gradual. Even in the phase damping noise environment, the anti-noise ability is improved by nearly 3 times. However, we also notice that in the first half of the bit-flip noise and phase-flip noise environments, the anti-noise ability of the scheme in this paper is not as obvious as in other noises. Therefore, in actual deployment, it is necessary to adjust the threshold specifically according to the characteristics of the transmission channel. In addition, in the key agreement process, in order to ensure that both the SM and the NAN gateway obtain the same key, redundant encoding can be considered or combined with classical check codes.

## 7. Conclusions

In view of the communication security problems among entities in the smart grid, this paper takes the Bell state as the quantum resource, draws on the idea of quantum private comparison, and proposes a complete identity authentication and key agreement scheme among power grid entities for the smart grid through Pauli operations and entanglement swapping. We prove that the proposed scheme has quantum security. Finally, we verify through experiments that the scheme in this paper has good noise resistance.

Just like classical cryptography, quantum cryptography can realize the secure transmission of data and ensure the privacy and security of data. However, quantum cryptography, by taking advantage of the characteristics of quantum mechanics, can further improve the security of classical cryptography, especially under the threat of quantum attacks. The supplement of quantum cryptography to classical cryptography can help to realize more complex and flexible protocols to deal with various practical problems.

## Figures and Tables

**Table 1:** Symbols and their explanations.

Symbols	Explanations
${ID}_{X}$	Identity of NAN gateway and smart meter
${PID}_{SM}$	Pseudonym of smart meter
$R_{X},R_{session}$	Random seed
H	Hash function family
$h_{K\sim X}(\cdot )$	Hash function
n	Output length of hash function, n is even
$F_{NAN}(\cdot )$	Key stream function
$K_{OTP\sim X}$	Key stream generated by random seed
TS	Timestamp
rand	Random number
$K_{session}$	Session key of NAN gateway and smart meter
$K_{SM}$	Master key between smart meter and the control center
$K_{NAN}$	Master key between NAN gateway and the control center
$B_{ij},M_{ij}$	The original state and measured state of Bell states during the identity authentication phase
$B_{ij}^{\prime },M_{ij}^{\prime }$	The original state and measured state of Bell states during the key agreement phase

**Table 2:** Integer generation.

$k_{N}$	Binary Representation	Decimal Representation
$k_{N1}$	011010	26 mod7
$k_{N2}$	110100	52 mod7
$k_{N3}$	101001	41 mod7
$k_{N4}$	010011	19 mod7
$k_{N5}$	100110	38 mod7
$k_{N6}$	001101	13 mod7

**Table 3:** Scheme comparison.

	Ref. [21]	Ref. [20]	Ref. [22]	Ref. [30]	Ours
Quantum source	Bell state	Bell state, Single photon	Single photon	GHZ state	Bell state
Quantum operations	Single particle measurement	Semi-quantum + Single particle Measurement	QKD + QRNG	Single particle measurement	Pauli operations + BSM
Classical operations	Hash + Symmetric Encryption	Hash + Symmetric Encryption	Hash + XOR	-	Hash + XOR
Quantum capabilities of participants *	Y	Y	Y	Y	N
Quantum efficiency	50%	50%, 11.1%	≈50%	100%	50%

*: The quantum capability of participants refers to whether other parties need to have the capability to prepare quantum states except for the CC.

**Table 4:** Noise and Kraus operators.

Noise Type	Kraus Operators
Amplitude damping noise	$E_{1}=\left[\begin{array}{cc} 1 & 0\\ 0 & \sqrt{1-p} \end{array}\right]$ $,\;E_{2}=\left[\begin{array}{cc} 0 & \sqrt{p}\\ 0 & 0 \end{array}\right]$
Phase damping noise	$E_{1}=\left[\begin{array}{cc} 1 & 0\\ 0 & \sqrt{1-p} \end{array}\right]$ $,\;E_{2}=\left[\begin{array}{cc} 0 & 0\\ 0 & \sqrt{p} \end{array}\right]$
Bit-flip noise	$E_{1}=\sqrt{1-p}\left[\begin{array}{cc} 1 & 0\\ 0 & 1 \end{array}\right]$ $,\;E_{2}=\sqrt{p}\left[\begin{array}{cc} 0 & 1\\ 1 & 0 \end{array}\right]$
Phase-flip noise	$E_{1}=\sqrt{1-p}\left[\begin{array}{cc} 1 & 0\\ 0 & 1 \end{array}\right]$ $,\;E_{2}=\sqrt{p}\left[\begin{array}{cc} 1 & 0\\ 0 & -1 \end{array}\right]$
Depolarizing noise	$E_{1}=\sqrt{1-p}\left[\begin{array}{cc} 1 & 0\\ 0 & 1 \end{array}\right]$ $,\;E_{2}=\sqrt{p/3}\left[\begin{array}{cc} 0 & 1\\ 1 & 0 \end{array}\right],\;$ $E_{3}=\sqrt{p/3}\left[\begin{array}{cc} 0 & -i\\ i & 0 \end{array}\right]$ $,\;E_{4}=\sqrt{p/3}\left[\begin{array}{cc} 1 & 0\\ 0 & -1 \end{array}\right]$

## Data Availability

The data presented in this study are available on request from the corresponding author.
